# Familial risk of systemic sclerosis and co-aggregation of autoimmune diseases in affected families

**DOI:** 10.1186/s13075-016-1127-6

**Published:** 2016-10-12

**Authors:** Chang-Fu Kuo, Shue-Fen Luo, Kuang-Hui Yu, Lai-Chu See, Weiya Zhang, Michael Doherty

**Affiliations:** 1 Division of Rheumatology, Orthopaedics and Dermatology, School of Medicine, University of Nottingham, NG51PD, Academic Rheumatology, Clinical Sciences Building, City Hospital, Nottingham, NG51PB Nottingham UK; 2 Division of Rheumatology, Allergy and Immunology, Chang Gung Memorial Hospital, Taoyuan, Taiwan; 3 Department of Public Health, College of Medicine and Biostatistics Core Laboratory, Molecular Medicine Research Centre, Chang Gung University, Taoyuan, Taiwan

**Keywords:** Systemic sclerosis, Familial aggregation, Familial transmission, Rheumatic diseases, Relative risk

## Abstract

**Background:**

Systemic sclerosis (SSc) is a rare and devastating disease affecting skin and internal organs. Familial aggregation of SSc and co-aggregation with other autoimmune diseases is rarely reported.

**Methods:**

We identified 23,658,577 beneficiaries registered with the National Health Insurance database in 2010, 1891 of whom had SSc. We identified 21,009,551 parent–child relationships and 17,168,340 full sibling pairs. The familial risks of SSc and other autoimmune diseases and familial transmission were estimated.

**Results:**

The prevalence of SSc in the general population was 0.008 %. There are 3801 individuals had at least one first-degree relative with SSc, among them 3 people had SSc which was equivalent to a prevalence of 0.08 %. The adjusted relative risk (RR) (95 % CI) for SSc was 81.21 (11.40–579.72) for siblings of SSc patients. The familial transmission (genetic plus shared environmental contribution to total phenotypic variance of SSc) was 0.72. However, 84.1 % of patients were expected to be sporadic cases. The RR (95 % CI) in first-degree relatives of SSc patients was 2.64 (1.46–4.75) for rheumatoid arthritis, 6.51 (4.05–10.46) for systemic lupus erythematosus, 2.77 (1.04–7.35) for Sjögren’s syndrome, 8.05 (2.03–31.92) for idiopathic inflammatory myositis, and 1.52 (1.15–2.01) for psoriasis.

**Conclusions:**

The risks of SSc and other autoimmune diseases are increased in relatives of people with SSc, and family factors explain over two-thirds of the phenotypic variance of the disease. These findings may be useful in counselling families of patients with SSc and for further genetic studies.

## Background

Systemic sclerosis (SSc) is an uncommon autoimmune disease characterized by the thickening of the skin and involvement of internal organs including blood vessels, heart, lung, kidney, and gastrointestinal tract. The prevalence and incidence of SSc are difficult to estimate because of its rarity and the difficulty to ascertain diagnosis. In the United States, the prevalence has been estimated at 24.2 cases per 100,000 adults [1]. In Taiwan, our group recently estimated the prevalence of SSc to be 5.6 per 100,000 people using the National Health Insurance (NHI) database, which contains health information of almost all the inhabitants in Taiwan [2, 3]. The outcome of SSc is generally thought to be poor, with a higher mortality and an increased risk for other severe comorbidity such as cancer [2–4].

SSc is thought to be a polygenic disorder which is contributed to by both genetic and environmental factors [5]. Evidence for familial aggregation has rarely been reported but indicates a possible familial or genetic contribution for SSc [6, 7]. The discovery of candidate genes located in both major histocompatibility complex (MHC) and non-MHC loci [8] has strengthened this concept; however, the relative contribution of genetic and shared environmental factors to SSc susceptibility (familial transmission) has not been adequately estimated. In addition, the precise estimates for familial relative risks (RRs) for the disease and other associated autoimmune diseases which are important parameters for genetic counselling have not been reported despite evidence for familial aggregation of Raynaud’s syndrome and an increased risk of SSc in individuals with a family history of systemic lupus erythematosus (SLE) [9, 10] and Sjögren’s syndrome [11].

Therefore, we conducted this nationwide study using genealogy and linked health information derived from the NHI database comprising essentially the entire population of Taiwan in 2010 to determine familial aggregation of SSc and to assess the relative contribution of familial factors to susceptibility for the disease. In addition, we also investigated the co-aggregation of other autoimmune diseases with SSc.

## Methods

### Study population

This study was based on a cohort comprising all beneficiaries of the Taiwan National Health Insurance (NHI) system actively enrolled in 2010, which was constructed using data from the registry for beneficiaries, registry for catastrophic illness patients, and datasets of ambulatory care expenditures and details of ambulatory case orders, all of which are part of the NHI database. The enrolment in the NHI system is mandatory by law for all citizens and foreigners living longer than 6 months in Taiwan and this is a single payer system managed by the government. In 2010, over 99.5 % of the general population in Taiwan was insured by the system [12]. The NHI database is updated annually and records information on personal details, socioeconomic status, family relationships, dates of clinical visits, medical diagnoses, medical expenditures, prescription details, examinations, and procedures. All beneficiaries are given a unique identification which allows precise internal linkage of all data for a given individual. This identification was encrypted before being released for research to ensure confidentiality but the uniqueness of the encrypted identification is retained to ensure valid internal linkage.

Methods of identification of first-degree relatives and family ascertainment have been reported previously [10, 11]. The relationships recorded in the NHI database include spouse, parent, offspring, and grandparents, grandchildren, great grandparent, and great grandchildren on both paternal and maternal sides. The identification of siblings was based on sharing one or more common parents. In this study, half-siblings were excluded from the analysis. Twins were full siblings with the same date of birth (±1 day) but twin zygosity cannot be derived from the database. To consider the correlation among subjects from the same family, we grouped individuals into families according to their relationships. In total, 21,009,551 parent–child relationships, 17,168,340 full sibling pairs and 342,066 twin pairs were identified and these relationships were used to assemble 4,229,301 families with a mean family size of five persons spanning up to five generations.

### Case definition of SSc and other autoimmune diseases

The case definition for SSc was a person with a catastrophic illness certificate for SSc (International Classification of Diseases, Ninth Revision (ICD-9) code 7101). The holders of a catastrophic illness certificate are entitled to a waiver of medical co-payments, which must be supported by comprehensive clinical and laboratory assessment. Relevant information is sent to the insurance administration for a formal review to confirm the diagnosis before approval of waivers. The panel reviews the diagnosis in compliance with the updated classification criteria. We also identified patients with other autoimmune diseases including rheumatoid arthritis, SLE, Sjögren’s syndrome, idiopathic inflammatory myositis, type I diabetes mellitus, and multiple sclerosis using the catastrophic illness registry. The case definition of psoriasis was based on two recordings of rheumatologist or dermatologist diagnosis.

### Statistical analysis

The prevalence of SSc was calculated for individuals with affected first-degree relatives and for the general population. Any individual with a valid insurance registration in 2010 who met the case definition of SSc between 1 January 1996 and 31 December 2010 was defined as a prevalent case. The number of individuals in the 2010 general population in Taiwan was used as the denominator for the prevalence of SSc. The recurrence risk for SSc was defined as the likelihood of having a diagnosis of SSc in an individual with affected first-degree relatives with a diagnosis of SSc. We calculated recurrence risk for a specific type of relative (sibling and offspring) of patients with SSc as the prevalence of SSc in individuals with a specific type of affected relative [13]. The adjusted prevalence ratio was used as a measure for relative risk (RR) for SSc [14], and was calculated as the prevalence of SSc among individuals with affected relatives divided by the prevalence of SSc in the general population. The RR estimated in this study is equivalent to the relative recurrence ratio (RRR) but for simplicity we refer it as RR throughout. The marginal Cox proportional hazard model with an equal follow-up time for all subjects was used to estimate the RR and the 95 % confidence interval (CI) [15]. We used the robust sandwich estimator to calculate corrected CIs to account for possible case clustering within the family [16]. This approach has been applied and validated previously in other diseases [17]. Covariates included age, gender, socioeconomic factors (place of residence, occupation, and income levels), and family size. A place of residence for each individual was categorised according to the level of urbanisation [18]. Occupations were classified into five categories and income levels were categorised into sex-specific income quintiles.

Heritability was defined as the proportion of phenotypic variance that is attributable to genetic factors, and familial transmission as the sum of heritability and the proportion attributable to shared environmental factors; both can be calculated using the polygenic liability model [19–21]. The familial transmission in this study was estimated as the function of the difference of normal deviation of the threshold from the mean liability between individuals with affected siblings and the normal population. The original model assumes zero common environmental variance and, therefore, familial transmission equals heritability. However, we consider this assumption not applicable in the case of SSc considering known environmental factors that may predispose to the disease and be shared among family members. In our previous paper, we further used spouses as a control to separate the shared environmental component and heritability [11]. As SSc is predominantly a female disease and its prevalence is low, it was not possible to identify enough affected spouse pairs to produce reliable estimates. Therefore, we only reported familial transmission. Next, we calculated the probability of a patient with SSc without a family history according to the formula based on a polygenic liability model developed by Yang et al. [22].

We further estimated the extent of familial co-aggregation of other autoimmune diseases in affected families. RRs and 95 % CIs for rheumatoid arthritis, SLE, Sjögren’s syndrome, idiopathic inflammatory myositis, type I diabetes mellitus, multiple sclerosis, and psoriasis were estimated as the adjusted prevalence ratio of specified autoimmune diseases between individuals with a first-degree relative with SSc and the general population. We estimated the RR for other autoimmune diseases by a marginal Cox proportional hazards regression model with an equal follow-up time for all subjects. The RRs were adjusted for age, sex, and family size, and considered case clustering within families by using the robust sandwich estimate.

A two-sided *p* value ≤0.05 was considered statistically significant. All analyses were performed using SAS v.9.3 (SAS institute, Cary, NC, USA).

## Results

Among 23,658,577 individuals enrolled in the NHI database in Taiwan in 2010, there were 1891 individuals (1495 women; 396 men) with SSc, equivalent to a prevalence of 0.008 % (women, 0.013 %; men, 0.003 %). In the general population of Taiwan in 2010, 3801 individuals had at least one first-degree relative with SSc. The baseline characteristics were shown in Table 1. Among people with a family history of SSc, three people had SSc which was equivalent to a prevalence of 0.08 %. Overall, having an affected first-degree relative with SSc was associated with an adjusted RR (95 % CI) of 13.23 (3.11–56.33) for the disease. For risks for specific kinship, RRs were 81.22 (11.40–578.72) for siblings and 9.17 (1.30–65.86) for offspring of patients with SSc. Using a polygenic liability model, we estimated that the familial transmission for SSc was 0.72. We estimated that 84.1 % of patients with SSc were expected to be sporadic cases.

Table 2 presents adjusted RR (95 % CIs) for other autoimmune diseases in individuals with affected first-degree relatives compared to the general population. The RR (95 % CI) in first-degree relatives of SSc patients was 2.64 (1.46–4.75) for rheumatoid arthritis, 6.51 (4.05–10.46) for SLE, 2.77 (1.04–7.35) for Sjögren’s syndrome, 8.05 (2.03–31.92) for idiopathic inflammatory myositis, and 1.52 (1.15–2.01) for psoriasis.

**Table 1:** Baseline characteristics of individuals with affected relatives with systemic sclerosis and the general population

Variables	≥1 Affected relatives	General population	*P* value
No.	3801	23,658,577	
Male gender (*n* (%))	2037 (53.59)	11,732,064 (49.59)	<0.001
Age (years) (mean ± SD)	46.6 ± 16.2	37.5 ± 20.4	<0.001
Systemic sclerosis (*n* (%))	3 (0.08)	1891 (0.008)	<0.001
Place of residence (*n* (%))			
Urban	2421 (63.69)	13,935,055 (58.90)	<0.001
Suburban	991 (26.07)	6,581,657 (27.82)	
Rural	267 (7.02)	2,186,647 (9.24)	
Unknown	122 (3.21)	955,218 (4.04)	
Income levels (*n* (%))			
Quintile 1	594 (15.63)	4,077,139 (17.23)	<0.001
Quintile 2	529 (13.92)	3,334,917 (14.10)	
Quintile 3	881 (23.18)	6,296,926 (26.62)	
Quintile 4	830 (21.84)	4,547,059 (19.22)	
Quintile 5	847 (22.28)	4,437,878 (18.76)	
Unknown	120 (3.16)	964,658 (4.08)	
Occupation (*n* (%))			
Dependents of the insured individuals	1185 (31.18)	9,209,334 (38.93)	<0.001
Civil servants, teachers, military personnel and veterans	195 (5.13)	984,451 (4.16)	
Non-manual workers and professionals	1426 (3752)	6,357,208 (26.87)	
Manual workers	631 (16.60)	4,885,084 (20.65)	
Other	364 (9.58)	2,222,500 (9.39)

**Table 2:** Relative risks of systemic sclerosis and other autoimmune diseases for individuals with affected first-degree relatives

Autoimmune diseases	With affected relatives		General population		Relative risk (95 % CI)^a^
	No. of cases	Prevalence (%)	No. of cases	Prevalence (%)	
Systemic sclerosis	3	0.08	1891	0.008	13.23 (3.11–56.33)
Rheumatoid arthritis	11	0.29	37,482	0.16	2.64 (1.46–4.75)
Systemic lupus erythematosus	20	0.53	18,915	0.08	6.51 (4.05–10.46)
Sjögren’s syndrome	4	0.11	12,754	0.05	2.77 (1.04–7.35)
Idiopathic inflammatory myositis	2	0.05	1808	0.01	8.05 (2.03–31.92)
Type I diabetes mellitus	5	0.13	10,281	0.04	2.51 (0.90–7.07)
Multiple sclerosis	1	0.03	1252	0.01	5.16 (0.73–3665)
Psoriasis	47	1.24	196,333	0.83	1.52 (1.15–2.01)

^a^ Adjusted for age, gender, place of residence, quintiles of income levels, occupations, and family size

## Discussion

This study investigated the risk of SSc in individuals with affected first-degree relatives and estimated the familial transmission of SSc in a general population. We found that the prevalence of SSc in relatives of patients with the disease is significantly higher than the general population and that the familial transmission of SSc was 0.72. Despite this, most cases of SSc are expected to be sporadic under the polygenic liability model. Furthermore, individuals with an affected first-degree relative with the disease also have a higher prevalence of other autoimmune diseases. These estimates are valuable for clinical counselling, and also for the preparation of future genetic studies to determine candidate susceptibility genes.

Formal evidence for familial aggregation and the magnitude of any familial or genetic contribution for SSc is rarely reported [6, 7, 23]. Englert et al. identified 710 Australian families with SSc and reported a prevalence of 1.4 % and a RR of 11 in people with affected family members [6]. Another study from the US reported a risk of 1.6 % in those with an affected first-degree relative and the RR was 13 [7]. The risk of SSc in an individual with an affected first-degree relative was estimated to be 1.1 %, which was 3.5-fold higher than the controls [23]. Another Canadian study reported that 2.8 % of individuals with a positive family history of SSc had the disease [24]. These studies in general indicate a high familial absolute and relative risk for SSc. However, the estimates vary over a wide range, probably due to variable study design, case ascertainment, and family member recruitment.

Estimates from both studies are similar to our study and indicate a high familiarity of SSc. Concordance of SSc in twins has been reported previously [25, 26]. In addition, monozygotic twins discordant for SSc were found to have a similar fibroblast gene expression pattern, suggesting a strong genetic predisposition to SSc at the molecular level in skin fibroblasts [27]. Interestingly, a small twin study comprising 24 monozygotic and 18 dizygotic twin pairs found a similar concordance rate for SSc between monozygotic (4.2 %) and dizygotic twin pairs (5.6 %). However, the concordance for a positive test for antinuclear antibody was very high (90 % in monozygotic and 40 % in dizygotic twins) [28]. In addition to twins, two reports have indicated familial forms of both diffuse cutaneous SSc [29, 30] and limited cutaneous SSc [31]. A positive family history of SSc is thought to represent the largest risk factor for this disease with a familial RR of 15 [7]. Using the Utah Population Database, the familial RR for SSc was approximately 2 to 3 in first- to third-degree relatives, but there is no apparent association between the magnitude of RR and genetic relatedness [32]. Heritability of SSc has not been assessed before; however, a twin study comprising 702 monozygotic and 727 dizygotic twin pairs reported a significant heritability (0.53) for Raynaud’s phenomenon [33].

Our study provides several lines of evidence supporting the importance of familial factors, including both genes and environment, in the susceptibility of SSc. Firstly, the prevalence of SSc was significantly higher in first-degree relatives of SSc patients than in the general population, in addition to a high familial RR. Secondly, using the polygenic liability model, we estimated that 72 % of phenotypic variance can be explained by familial factors (familial transmission). Despite this apparent familial factor in the susceptibility of SSc, most patients are expected to be sporadic rather than familial under the polygenic liability model as with many common complex diseases [11, 22]. For example, 78–84 % of patients with rheumatoid arthritis, SLE [22], and Sjögren’s syndrome [11] are expected to be sporadic cases depending on the parameters used [22].

In addition to SSc, other autoimmune diseases are more common in family members of patients with the disease, which suggest that some autoimmune diseases share part of the pathogenesis of SSc. Previous studies have documented that autoimmune diseases often co-aggregate within families. Several studies also found an increased risk of other autoimmune disease in family members of SSc patients [9–11, 23, 24, 34, 35]. A relatively large study investigating 4612 first-degree relatives of 1071 SSc probands found that the most prevalent autoimmune diseases among first-degree relatives of SSc patients were hypothyroidism, rheumatoid arthritis, hyperthyroidism, and SLE [34]. Collectively, our results provide useful information when counselling patients and their family members in which circumstances and details of genetic parameters related to SSc should be given and explained in full. An unselective screening for SSc and other autoimmune diseases in all asymptomatic family members of patients with SSc is not recommended given a low absolute risk of these conditions. On the contrary, a positive family history is an important hint for those suspected of having an autoimmune disease. However, further study should be undertaken to test the utility of family history as a tool to identify at-risk individuals.

### Limitations and strengths

There are limitations to the present study. Firstly, the classification of cases was based on the diagnosis recorded in the registry of patients with catastrophic illnesses or based on records in primary care. Nevertheless, issuance of a catastrophic illness certificate requires strong medical evidence for a diagnosis of SSc that is agreed by an expert panel, and applications for these certificates are submitted almost exclusively by rheumatologists. Therefore, any misclassification is unlikely to affect our conclusions. However, some patients with milder disease may not have applied for the certificate and this might affect our results. Secondly, we are not able to differentiate different forms of SSc. Thirdly, our model cannot effectively separate contributions from genetic and shared environmental factors. Finally, whether these results apply to different populations and settings outside of Taiwan requires further study.

## Conclusions

This nationwide study confirms that, in Taiwan, SSc clusters within families and familial factors contribute to a large proportion of the susceptibility to the disease. Relatives of patients with SSc tend to have an increased risk of other autoimmune diseases. These findings may also be useful in counselling families with patients with SSc.

## Abbreviations

CI: Confidence interval
MHC: Major histocompatibility complex
NHI: National Health Insurance
RR: Relative risk
SLE: Systemic lupus erythematosus
SSc: Systemic sclerosis
